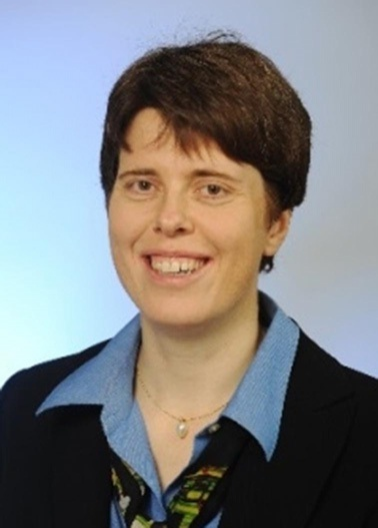# Low‐Temperature Miniemulsion‐Based Routes for Synthesis of Metal Oxides

**DOI:** 10.1002/chem.202001246

**Published:** 2020-07-09

**Authors:** Rafael Muñoz‐Espí, Katharina Landfester

**Affiliations:** ^1^ Institute of Materials Science (ICMUV) Universitat de València c/ Catedràtic José Beltrán 2 46980 Paterna Spain; ^2^ Max Planck Institute for Polymer Research Ackermannweg 10 55128 Mainz Germany

**Keywords:** confinement, crystallization, inorganic, miniemulsion, oxide

## Abstract

The use of miniemulsions containing chemical precursors in the disperse phase is a versatile method to produce nanoparticles and nanostructures of different chemical nature, including not only polymers, but also a variety of inorganic materials. This Minireview focuses on materials in which nanostructures of metal oxides are synthesized in processes that involve the miniemulsion technique in any of the steps. This includes in the first place those approaches in which the spaces provided by nanodroplets are directly used to confine precipitation reactions that lead eventually to oxides. On the other hand, miniemulsions can also be used to form functionalized polymer nanoparticles that can serve either as supports or as controlling agents for the synthesis of metal oxides. Herein, the description of essential aspects of the methods is combined with the most representative examples reported in the last years for each strategy.

## Graphical abstract

**Emulsions**: This minireview focuses on the use of miniemulsion‐based systems for the preparation of metal oxide nanostructures, including those nanoparticles prepared within the nanoconfinement of nanodroplets, but also extending the overview to polymer nanoparticles prepared by miniemulsion polymerization, serving either as supports or as controlling agents for the synthesis of metal oxides.

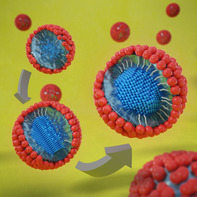

## 1 Introduction

Droplets of a liquid dispersed in another immiscible liquid form colloidal systems commonly known as emulsions. If such droplets contain chemical reagents, they are able to confine spaces in which chemical processes can occur, serving as soft templates for the formation of nanostructures.^1^ In an ideal case, if coalescence of the droplets and Ostwald ripening are suppressed—or at least minimized—each droplet can act as an independent “nanoreactor”.^2^, ^3^ This situation takes place, indeed, in so‐called miniemulsions, which have been widely used for the preparation of polymer particles,^3^, ^5^ but also for the formation of hybrid and inorganic materials.^6^, ^9^ The confinement can occur not only within droplets, but also at the liquid–liquid interface,^10^ which is interesting for preparing nanocapsules.

Before entering in the details of synthesis of specific materials, it is necessary to clarify the concept of miniemulsion, because the terminology used for emulsion systems is not especially logical, and may be confusing for an outsider of the field. Following a commonly used classification,^11^ based on the size of the droplets and the stability in time, emulsions can be classified in macroemulsions (with droplets typically larger than 1 μm and short stability up to minutes or hours), miniemulsions (with droplets between 50 and 500 nm and stability from days to months), and microemulsions (with droplets below 100 nm and thermodynamically stable). A first confusing issue may be the fact that, in spite of what the name could suggest, microemulsions have sizes clearly below the micron size. The term miniemulsion has its origins in the polymer community, in the context of the miniemulsion polymerization, contrasting to conventional emulsion polymerization, and it has been typically linked not only to droplet size, but also to mechanistic aspects, such as the use of high‐shear forces for kinetic stabilization and the important presence of osmotic pressure agents in the system. To complicate more the terminology, the label nanoemulsion (intentionally hyphenated as nano‐emulsion by some authors^12^, ^13^) is also found in literature, mostly as a “quasi‐synonym” of miniemulsion, but apparently free of the mechanistic implications imposed to the definition of the latter. The term nanoemulsion, clearly more recent than miniemulsion, has become relatively frequent in the pharmaceutical field and food science in the last years.^14^, ^15^, ^16^ This name seems to be purely based on size, so that, in a broad sense, any emulsion with droplets in the nanometric scale could be theoretically considered as a nanoemulsion. In practice, however, the most common is to keep microemulsions (thermodynamically stable systems) as a differentiated case in any classification and reserve the term nanoemulsion for metastable systems. In our case, following the tradition of our own school, we speak here about miniemulsions, the most established term from a historic point of view. Nevertheless, in most of the situations, the term miniemulsion can be exchanged here with nanoemulsion.

In this Minireview, we focus very specifically on the use of miniemulsion‐based systems for the preparation of metal oxide materials, including first those nanoparticles that are strictly prepared within the confinement of nanodroplets, but also extending the overview to metal oxides supported on other materials prepared by miniemulsion (i.e., metal oxides crystallized on the surface of polymer nanoparticles resulting from miniemulsion polymerization). Finally, because of the affinity with the topic, we will also shortly refer to the use of miniemulsion‐based materials, namely functionalized latex particles, as additives or controlling agents in the synthesis of metal oxides.

## 2 Miniemulsions for the Synthesis of Metal Oxide Nanoparticles

The formation of inorganic nanoparticles starting from miniemulsions of precursors was reviewed in detail a few years ago.^6^ In the meantime, other works have also partially addressed the topic.^1^, ^17^, ^18^, ^19^ In this section, we aim to focus specifically on the use of miniemulsion droplets as “nanoreactors” for the confinement of precipitation reactions leading to the formation of crystalline metal oxides.

When speaking about the use of water‐in‐oil droplets for inorganic synthesis, the work of Pileni and her team is a compulsory reference, although they focused almost exclusively on thermodynamically stable systems, that is, on microemulsions.^20^, ^21^, ^22^ Nevertheless, in spite of the differences between microemulsions and miniemulsions, the idea behind in terms of confinement is essentially the same: a metal precursor is contained in droplets dispersed in an immiscible continuous phase. At least theoretically, chemical systems prepared in microemulsion can be analogously prepared in miniemulsion. However, it is important to note that in miniemulsions the identity of the droplets does not change with time. Therefore, a reaction within the droplet is indeed confined to the same droplet. A reaction between droplets is only possible if the droplets are forced to combine by applying shear. In microemulsions, the identity of droplets is not maintained, leading to a fast interchange of materials between droplets. Although the use of water‐soluble precursors in the disperse phase of inverse emulsions is the most common, oil‐soluble precursors can also be applied in direct (oil‐in‐water) systems.^23^, ^24^, ^25^, ^26^ For the works dealing with microemulsions, the reader is referred to specific reviews on the topic.^24^, ^27^, ^28^, ^29^, ^30^ For miniemulsions dealing with metal oxide and oxidic‐related materials, we have compiled the most relevant works of the last years in chronological order in Table 1.

**Table 1:** Metal oxide systems prepared up to date by inverse miniemulsion. The works are listed in chronological order of publication.

System	Precursor	Continuous Phase	Surfactant	References
Fe_2_O_3_	FeCl_2_ **⋅**4 H_2_O	Cyclohexane or Isopar M	P(S/EO)^[a]^	31
TiO_2_	TIP^[b]^	Isopar M	P(E/B‐*b*‐EO)^[c]^	32
Zr_*x*_Ti_1−*x*_O_2_, ZrTiO_4_	Zr(O^*i*^Pr)_4_ **⋅** ^*n*^PrOH and TIP^[b]^	Isopar M	P(E/B‐*b*‐EO)^[c]^	33
CeO_2_	Ce(NO_3_)_3_ **⋅**6 H_2_O	Cyclohexane	P(E/B‐*b*‐EO)^[c]^ and PIBSP^[d]^	34
Fe_2_O_3_	FeCl_3_	*n*‐Decane	Glissopal EM‐23	35
HfO_2_, ZrO_2_, HfZr_1−*x*_O_2_	ZrOCl_2_ **⋅**8 H_2_O and HfOCl_2_ **⋅**8 H_2_O	Toluene	PIBSP^[d]^	36
ZnO	ZnSO_4_	*n*‐Decane	Glissopal EM‐23	37
ZnO and M:ZnO^[e]^	Zn(NO_3_)_2_ **⋅**6 H_2_O	Cyclohexane	Triton X100	38, 39
YCrO3	Cr(NO_3_)_3_ **⋅**9 H_2_O and YCl_3_	Toluene	PIBSP^[d]^	40
Au/TiO2	Single source precursor^[f]^	Pentanol/Heptane	SDS^[g]^ and Triton X100	41
ZnO	ZnSO_4_	*n*‐Decane	PGPR^[h]^ and Span 80	42
CuO	Cu(NO_3_)_2_ **⋅**3 H_2_O	Toluene	PIBSP^[d]^ and PGPR^[h]^	43
ZnO	Zn(AcO)_2_ **⋅**2 H_2_O	*n*‐Decane	Span 20	44
CeO_2_, Fe_2_O_3_	Ce(NO_3_)_3_ **⋅**6 H_2_O, FeCl_2_, and FeCl_3_ **⋅**6 H_2_O	Toluene	PIBSP^[d]^, PGPR^[h]^, and P(S‐*b*‐AA)	45
Ce_1−*x*_Cu_*x*_O_2_	Cu(NO_3_)_2_ **⋅**3 H_2_O and Ce(NO_3_)_3_ **⋅**6 H_2_O	Toluene	PGPR^[h]^	46
Fe_3_Mn_3_O_8_, Fe_3_O_4_, MFe_2_O_4_^[j]^	MCl_2_ **⋅** *n* H_2_O	Cyclohexane	PGPR^[h]^	47, 48

a) P(S/EO): poly(styrene/ethylene oxide); b) TIP: titanium isopropoxide; c) P(E/B‐*b*‐EO): poly(ethylene/butylene‐*block*‐ethylene oxide); d) PIBSP: polyisobutylene succinimide pentamine; e) M=Ag^I^, Co^II^, Cu^II^, Eu^III^, Mg^II^, Mn^II^; f) Single source precursor: AuCl_4_(NH_4_)_7_[Ti_4_(O_2_)_4_(cit)(Hcit)_2_]_2_⋅12H_2_O; g) SDS: sodium dodecyl sulfate; h) PGPR: polyglycerol polyricinoleate; i) P(S‐*b*‐AA): poly(styrene‐*block*‐acrylic acid); j) M: Co^II^, Cu^II^, Ni^II^, Zn^II^.

Some of the systems (e.g., Fe_2_O_3_, CeO_2_, CuO) can be obtained in the form of crystalline metal oxides already at low temperatures close to room temperature,^43^, ^45^ while other systems, typically obtained through conventional sol–gel routes (e.g., TiO_2_, ZrO_2_, HfO_2_), require calcination steps to reach crystals from the amorphous hydroxogels obtained in miniemulsion.^32^, ^36^ The high temperature requirement is intrinsic to these sol–gel systems and it is not exclusive to miniemulsion. Any method dealing with these systems will face the same temperature needs to reach crystalline materials.

Calcination steps have also been applied sometimes for particles containing inorganic metal precursors (e.g., cerium(III) nitrate to obtain CeO_2_ 34) or encapsulating organometallic compounds (e.g., organotin compounds to obtain SnO_2_ 49). We mention these examples here, since they are prepared in miniemulsion, but we do not enter in further details, as they cannot be considered “low‐temperature” routes.

**Figure 1:**
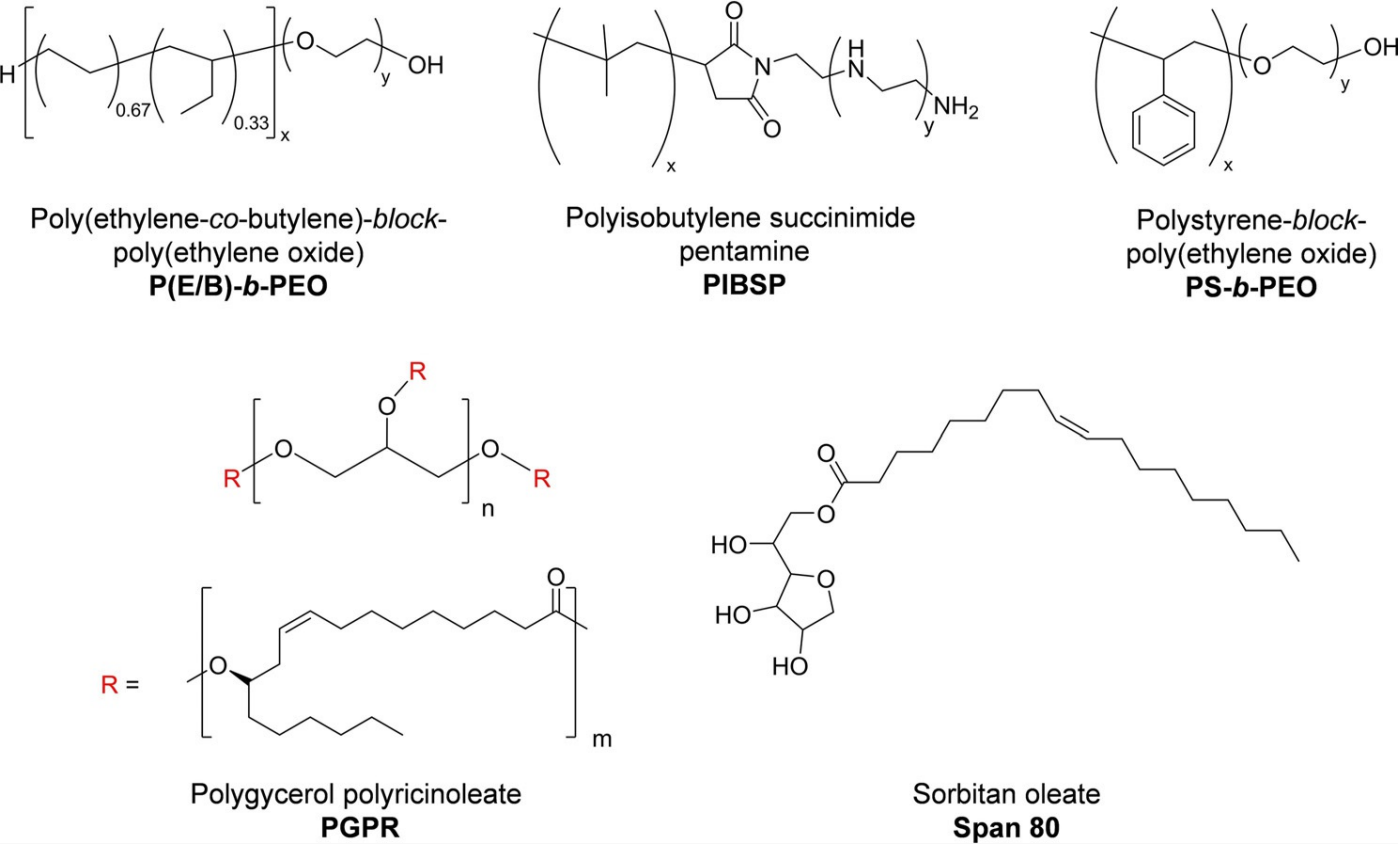
Chemical structure of some common surfactants in inverse miniemulsions.

Typical apolar solvents in inverse miniemulsion are cyclohexane (b.p.: 80.7 °C, which allows for easy evaporation after preparation of the oxidic particles), toluene (b.p.: 110.6 °C) or oils such as *n*‐decane and Isopar M (b.p. >150 °C, which allows for higher synthesis temperatures, but it makes the removal of the solvent afterwards difficult). The stabilization of inverse (water‐in‐oil) systems is always more challenging than the stabilization of direct (oil‐in‐water) ones. As surfactants, compounds with a low hydrophilic–lipophilic balance (HLB) are typically required (values <7 are common), although exceptions to this general rule can sometimes be found. The chemical structures of some common surfactants for inverse miniemulsions are presented in Figure 1. Block copolymers such as poly(styrene‐*block*‐ethylene oxide) (P(S/EO)) or poly(ethylene‐*co*‐butylene)‐block‐poly(ethylene oxide) (P(E/B‐*b*‐EO)) have been efficiently used (see Table 1). However, these types of copolymers are synthetically complex and usually not easily available from commercial sources. In this sense, commercial alternatives, such as polyisobutylene succinimide pentamine (PIBSP) and polyglycerol polyricinoloeate (PGPR), are quite convenient. It should be mentioned though that the amine groups of PIBSP can strongly interact with certain ions such as Cu^2+^,^43^ which may or may not lead to desirable effects. On the other hand, PGPR, an emulsifier used in food technology, is unfortunately not always able to efficiently stabilize some systems containing metal ions.

**Figure 2:**
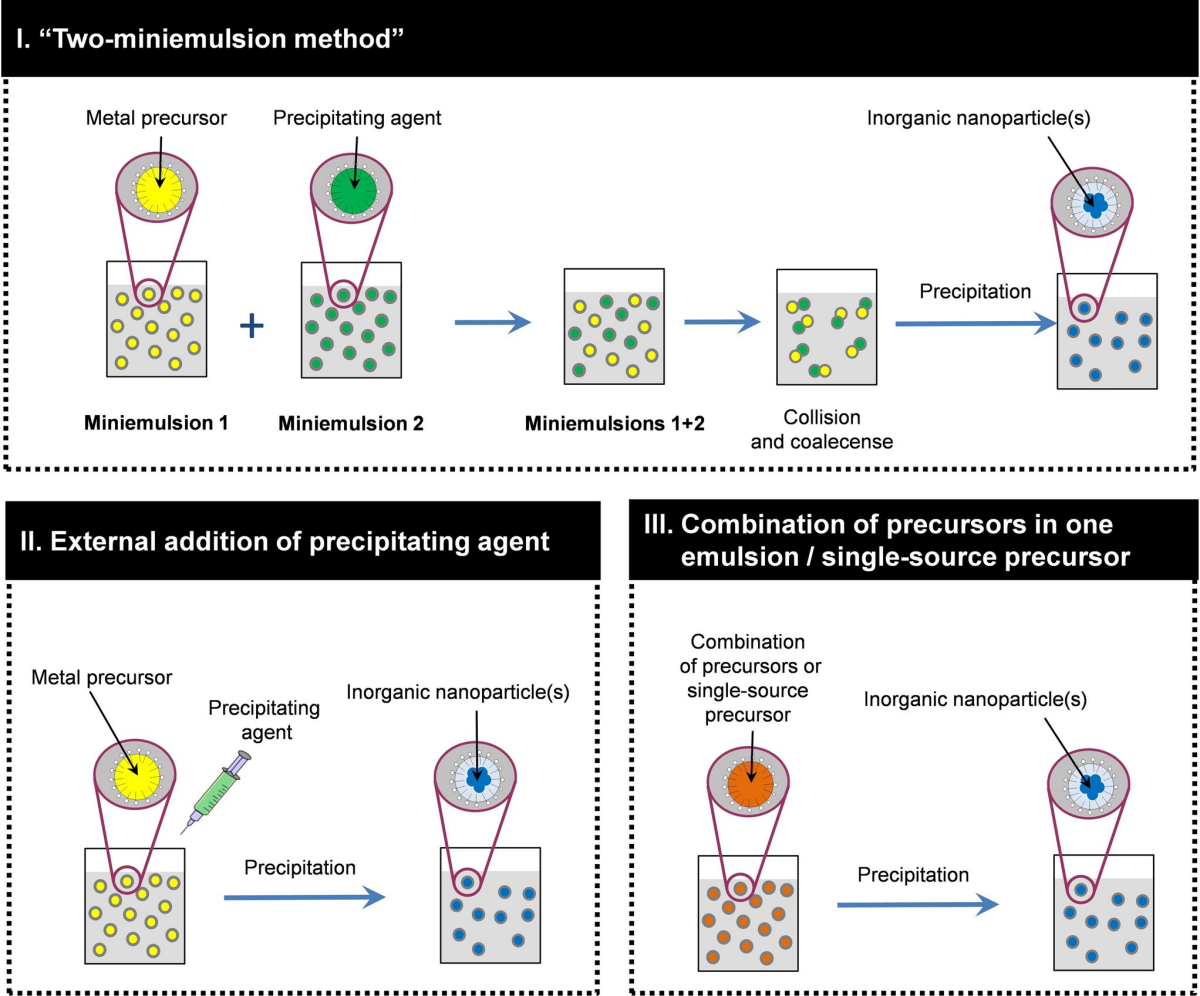
Schematic representation of the different approaches to prepare metal oxide nanoparticles from miniemulsions of metal precursors.

The first example of the use of miniemulsions for inorganic synthesis, including the case of Fe_2_O_3_, was reported by Willert et al.^31^ The initial approach was to use molten salts dispersed in an immiscible solvent. The molten salts recrystallize when the temperature is decreased. This strategy, however, cannot be directly used for the case of metal oxides, since the melting point of oxides is much above the operating temperature of any organic solvent. Therefore, a precipitation reaction or a sol–gel process is always involved in the formation of metal oxides in miniemulsions. In an attempt of systematization, we classify the existing possibilities in three different approaches, which are schematically depicted in Figure 2: I) “two‐miniemulsion methods”, II) external addition of a precipitating agent, and III) combination of precursors in the disperse phase or, alternatively, use of single‐source precursors.

**I. “Two‐miniemulsion methods”**: In this strategy, two independent miniemulsions containing the metal precursor (first component) and the precipitating agent (second component) are mixed, forcing the droplets of both components to coalesce and react, so that metal hydroxide species precipitate, turning eventually into the oxide. This method has been used, for instance, to prepare ZnO and doped‐ZnO nanoparticles by mixing miniemulsions containing a zinc salt and a base, such as NaOH or ammonia.^38^, ^39^, ^42^ In comparison, in microemulsions, the reactions between the different species take place instantaneously and less controlled without applying extra shear.^1^

**II. External addition of a precipitating agent to an emulsion of the metal precursor**: The precipitating agent (normally a base that generates OH^−^) can be water‐soluble or oil‐soluble. If working with inverse systems (water‐in‐oil), which is the most common for metal oxides, the addition of a base soluble in the continuous phase, such as triethylamine, can lead to the formation of hollow nanostructures. This effect, involving an interfacial precipitation/crystallization, has been reported for sol–gel systems such as zirconia and hafnia.^36^ Zirconium or hafnium oxychloride is dissolved in water and dispersed in an oil phase. Afterward, triethylamine (Et_3_N) is added to the system. In contact with the water of the droplets, Et_3_N generates hydroxide ions [Equation (1)]:

(1) $$\mathrm{Et}{}_{3}N+H{}_{2}O\vec{\leftarrow }\mathrm{Et}{}_{3}\mathrm{NH}{}^{+}\;\mathrm{OH}{}^{-}$$

The oxychloride precursor (MOCl_2_ **⋅** *n* H_2_O) reacts with the hydroxide ions to form hydrous zirconia or hafnia (MO(OH)_2_ **⋅** *n* H_2_O, M=Zr, Hf) [Equation (2)]:

(2) $$2\;\mathrm{Et}{}_{3}\mathrm{NH}{}^{+}\;\mathrm{OH}{}^{-}+\mathrm{MOCl}{}_{2}\vec{\leftarrow }\mathrm{MO}\left(\mathrm{OH}\right){}_{2}+2\mathrm{Et}{}_{3}\mathrm{NH}{}^{+}\;\mathrm{Cl}{}^{-}$$

The presence of hydroxide ions catalyzes the condensation reaction of the metal hydroxo species, as occurs in a sol–gel process. As above indicated, the final oxide formation requires a calcination step. This methodology has certain parallelism to the one reported in other works for preparing silica capsules in inverse miniemulsions,^50^, ^51^, ^52^, ^53^ in which an alkoxysilane is added through the continuous phase to an inverse miniemulsion. When the alkoxysilane enters in contact with the water of the droplets, it hydrolyzes and starts a condensation process.

In a similar fashion, but with conventional precipitation reactions without sol–gel, the preparation of CuO,^43^ ZnO,^37^, ^44^ CeO_2_,^45^ and Fe_2_O_3_ 35, 45 was also reported. In all these cases, oil‐soluble amines (i.e., triethylamine‐which is also partially soluble in water‐or the more apolar oleylamine) were used as a precipitating base. The contact of the OH^−^ ions with the metal ions contained in the droplets starts at the interface, which may lead to capsular morphologies. The shell formation may be assisted by the presence of surfactants at the interface such as poly(styrene‐*block*‐acrylic acid)^45^ or polyisobutylene succinimide pentamine (PIBSP),^43^ able to complex metal ions and act as structuring agents. Interestingly, for these systems, the materials are already crystalline when the synthesis is carried out at temperatures as low as room temperature. The combination of different metal precursors during the synthesis in defined amounts allows for obtaining ternary or doped oxides (e.g., YCrO_3_ or Ce_1−*x*_Cu_*x*_O_2_).^40^, ^46^

If the added base is water‐soluble (e.g., NaOH, KOH, or ammonia) and added as an aqueous solution, it is not miscible with the continuous phase, and an additional homogenization step (strong stirring or a further ultrasonication step) is required to induce the contact between the metal precursor and the precipitating agent.^47^, ^48^

**III. Mixture of precursors in the disperse phase or use of single‐source precursors**: When the reaction between the precursor and the precipitating agent is not very fast or requires some stimulus, such as temperature, they can be combined in the disperse phase of a single miniemulsion, which is subsequently left to react. This situation is the case of sol–gel systems, such as TiO_2_. Rossmanith et al.^32^ reported how inverse miniemulsions containing a titanium alkoxide in aqueous solution in the disperse phase led under acidic catalysis to TiO_2_ nanoparticles. Zirconium‐doped anatase (Zr_*x*_Ti_1−*x*_O_2_) could be prepared in an analogous way.^33^

For some systems, it is possible to obtain the oxide starting from one single precursor, a so‐called single‐source precursor. One example of this case is the formation of molybdic acid (hydrated forms of molybdenum trioxide, MoO_3_ **⋅** *n* H_2_O) from peroxo‐complexes [Equations (3), (4), (5)]:^54^

(3) $$H{}_{2}\mathrm{Mo}{}_{2}O{}_{3}\left(O{}_{2}\right){}_{4}\vec{\leftarrow }2\;\mathrm{MoO}{}_{3}\;1/2H{}_{2}O+2\;O{}_{2}$$

(4) $$2\;H{}_{2}\mathrm{MoO}{}_{2}\left(O{}_{2}\right){}_{2}\vec{\leftarrow }2\;\mathrm{MoO}{}_{3}\;H{}_{2}O+O{}_{2}$$

(5) $$2\;\mathrm{MoO}{}_{2}\left(\mathrm{OH}\right)\left(\mathrm{OOH}\right)\vec{\leftarrow }2\;\mathrm{MoO}{}_{3}\;H{}_{2}O+O{}_{2}$$

Such peroxo‐complexes can be obtained by reacting molybdenum metal with hydrogen peroxide,^55^ and have also been investigated in miniemulsion to obtain molybdic acid and, in an analogous way, tungstic acid and mixtures of these hydrated oxides.^56^

Another example of the use of a complex single‐source precursor in miniemulsion was reported by Heutz et al.,^41^ who prepared Au/TiO_2_ materials starting with a gold‐ containing titanium peroxo‐complex with structure AuCl_4_(NH_4_)_7_[Ti_4_(O_2_)_4_(cit)(Hcit)_2_]_2_ **⋅**12 H_2_O.

In general, the miniemulsion technique mainly allows us to reach spherical shapes. However, this does not always apply to inorganic materials, since the droplet size in the precursor emulsion is typically larger than the final inorganic particles obtained, so that the shape is not templated “one‐to‐one”, and other morphologies are possible. In addition, non‐spherical shapes are sometimes reachable under certain conditions, such as higher concentration of surfactant, which may lead to cylindrical micelles in the initial emulsion and, consequently, to rod‐like morphologies of the metal oxides.^56^

So far, most of the investigations about the use of miniemulsions for confining crystallization processes have been carried out under ambient pressure. The combination of hydrothermal conditions (i.e., not only temperatures above room temperatures, but also pressures above ambient conditions) with the miniemulsion technique have a very wide and exciting range of possibilities for the morphosynthetic control of amorphous and crystalline materials. In recent work, the synergy of hydrothermal and miniemulsion conditions was investigated for the preparation of different nanostructured metal ferrites.^47^ Different spinel ferrites, including Fe_3_MnO_8_, CoFe_2_O_4_, CuFe_2_O_4_, NiFe_2_O_4_, and ZnFe_2_O_4_ were prepared by adding a base (NaOH) to an inverse emulsion of the metal precursors (metal salts) and sonicated again to allow coalescence and precipitation according to method II. This work demonstrated that for some of the systems (namely, the zinc ferrites), the analogous materials obtained with miniemulsion at ambient pressure and under bulk conditions either at ambient pressure or under solvothermal conditions did not result in comparatively highly crystalline ferrites, thus outlining the relevance of the combined synthetic strategy.

## 3 Miniemulsions for the Synthesis of Supporting Materials for Metal Oxides

**Figure 3:**
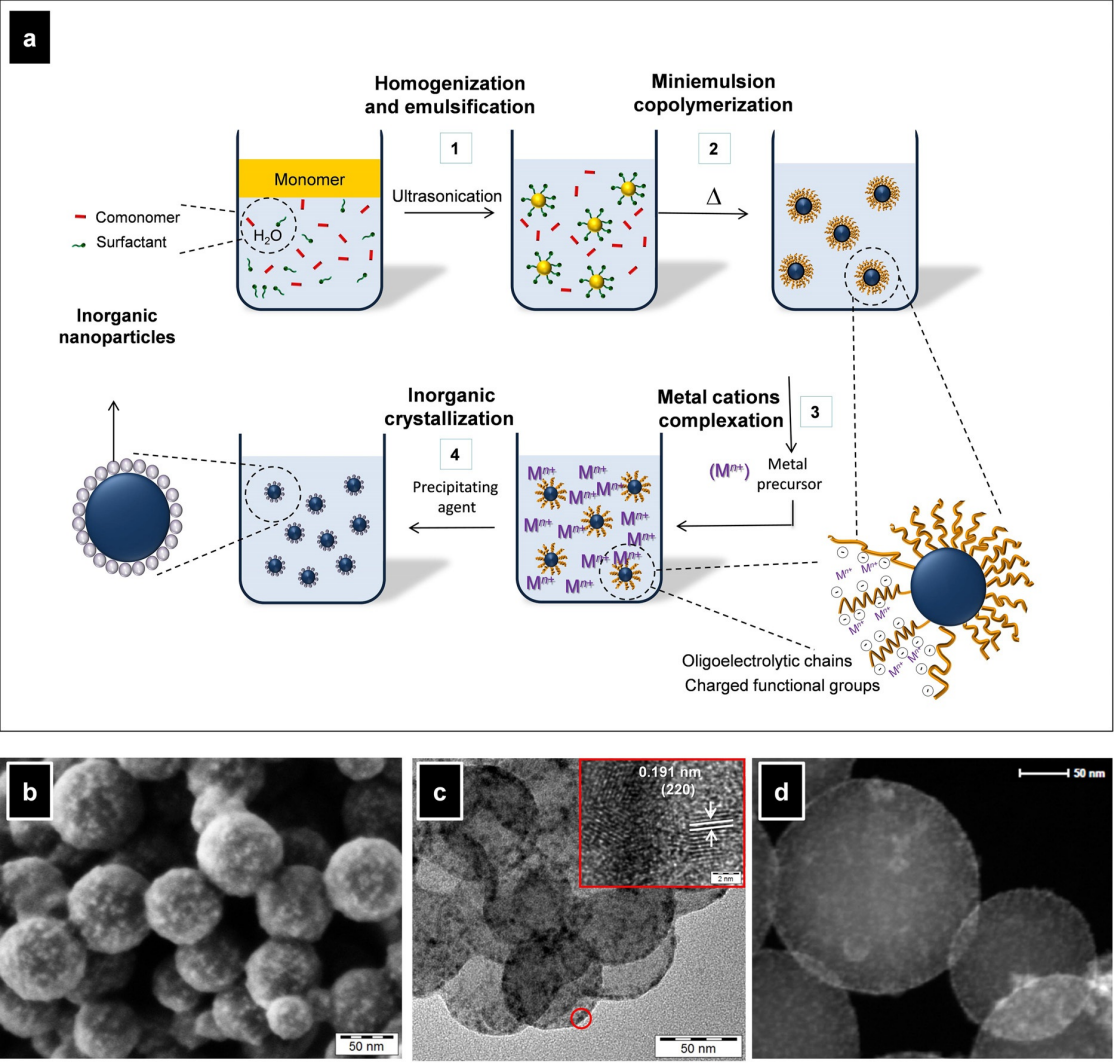
(a) Schematic representation of the formation of metal oxide nanocrystals on the surface of functionalized polymer nanoparticles prepared by miniemulsion polymerization. (b) SEM, (c) TEM, and (d) dark‐field TEM images of CeO_2_/poly(styrene‐*co*‐acrylic acid) hybrid nanoparticles prepared by this method. Adapted with permission from ref. 58. Copyright 2015, American Chemical Society.

The synthesis of metal oxide nanostructures can be confined not only within droplets, as described in the previous section, but also on the nanometric surfaces of nanoparticles of another material. In this context, functionalized polymer nanoparticles have been widely used for supporting the in situ growth of different inorganic crystals, including metal oxides (see a detailed overview in Ref. 1). Herein, we refer briefly to the examples dealing exclusively with polymer particles prepared by miniemulsion polymerization. The steps of the approach, together with some representative micrographs of the materials obtained, are presented in Figure 3. Carboxylates, phosphates, and phosphonate groups have been shown to be effective nucleating agents for the subsequent precipitation of metal oxide nanoparticles. The introduction of these functional groups on the surface of polymer nanoparticles can be achieved by copolymerization in miniemulsion of a supporting structural monomer (such as styrene or methyl methacrylate) with functional monomers (typically hydrophilic) that contain the desired metal complexing group. To avoid the use of surfactants, which may involve undesired effects in some cases, the use of so‐called surfmers (i.e., polymerizable surfactants or *surf*ace active mono*mers*) have been proposed.^57^ A metal precursor (usually a water‐soluble metal salt) is added to a dispersion of the surface‐functionalized polymer nanoparticles. The surface should have the ability to bind the metal cations, creating centers in which the nucleation of the metal oxide starts upon addition of the precipitating agent. The method is versatile for any metal oxide involving precipitation by addition of a base. Reported examples are CeO_2_, Fe_2_O_3_, Fe_3_O_4_, and ZnO. The support with this strategy of catalytic oxides, such as CeO_2_, leads to highly efficient and easily separable heterogeneous catalysts.^57^, ^58^

If the supporting material possesses a capsular morphology, which is also possible by employing miniemulsion polymerization, the resulting material can encapsulate further substances, in addition to the metal oxide on the surface. This methodology has been recently used to prepare hybrid nanocapsules containing CeO_2_ on the surface and organic fluorophores in the core.^59^, ^60^

## 4 Miniemulsions for the Synthesis of Controlling Agents for the Crystallization of Metal Oxides

The use of large organic molecules and polymers as templating and controlling agents in inorganic synthesis is a common strategy inspired by biomineralization processes in nature. Although metal oxides are rather unusual in biomineralization, there are a few examples of iron and manganese oxides in bacteria.^61^ In materials science, the use of polymers in crystallization has been often termed as “polymer‐controlled crystallization”,^62^ and it may be linked to so‐called “non‐classical crystallization” processes involving the formation of mesocrystals.^63^, ^64^ A review of the topic is clearly beyond the scope of this paper, but we consider appropriate at this position to refer to the few specific examples of miniemulsion polymers used to control crystallization of metal oxides.

**Figure 4:**
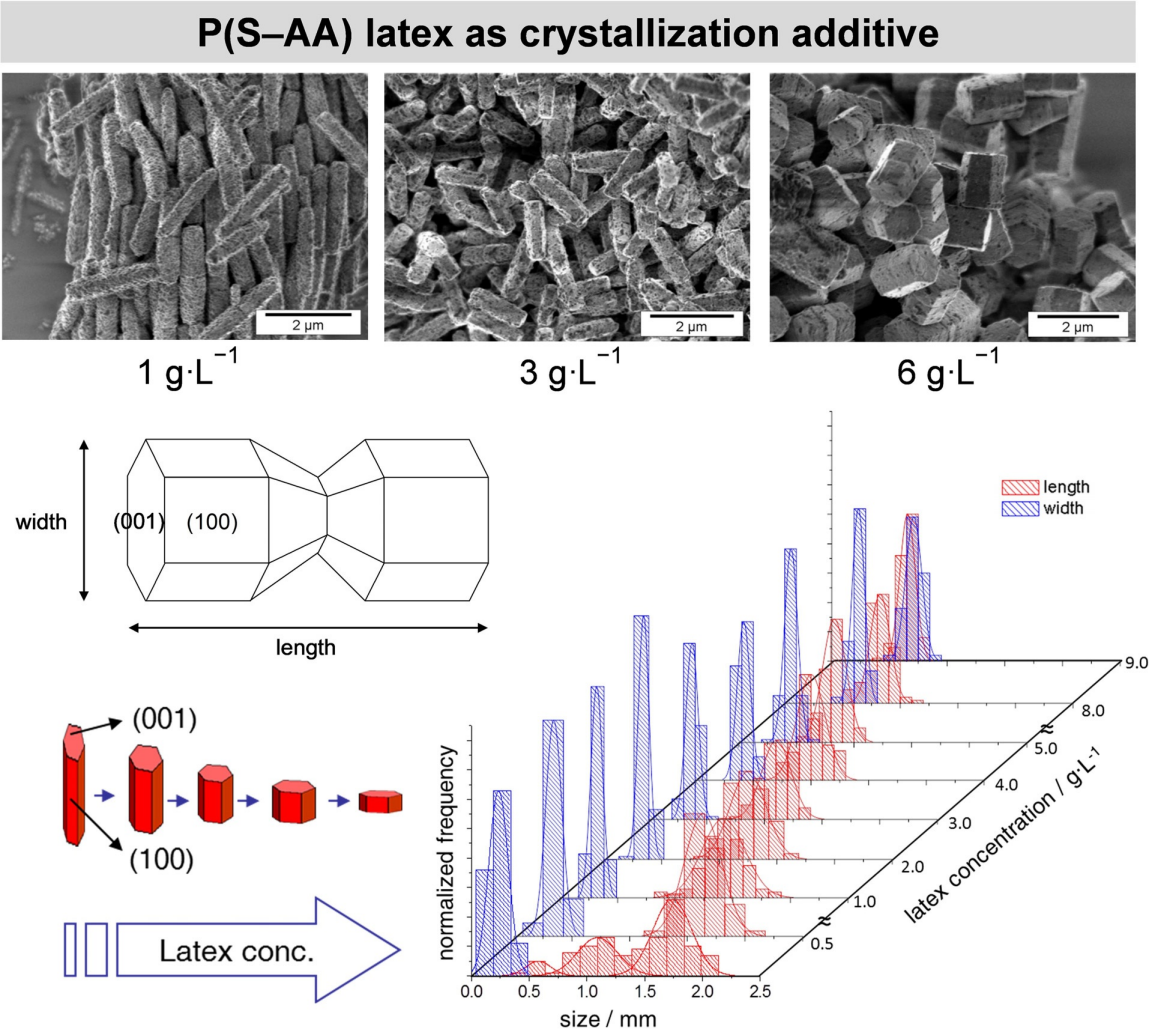
Change in the aspect ratio (length/width) of zinc oxide crystals prepared in the presence of different concentrations of poly(styrene/acrylic acid) nanoparticles synthesized by miniemulsion polymerization. SEM images and histograms of the lengths and widths of the prepared crystals. Adapted with permission from ref. 68. Copyright 2006, Wiley‐VCH.

In a seminal work by Wegner and co‐workers,^65^ zinc oxide was synthesized in the presence of poly(styrene/acrylic acid) latex particles prepared by miniemulsion copolymerization. It was found that the polymer nanoparticles became incorporated into the crystals, leading to what the authors named a “Swiss cheese morphology”.^66^ In the following years, a more detailed investigation of the mechanism of these systems followed (Figure 4).^67^, ^68^ Zinc oxide, with only one crystal phase under normal conditions (namely, zincite), was found to be a very suitable model for studying the change in morphology as a result of the presence of additives. The ability of miniemulsion copolymerization to deliver particles with different functional groups at the surface and with controlled surface charge density was further exploited for the zinc oxide system. Negatively charged polymer particles were found to adsorb preferentially on the {0 0 1} facets, retarding the growth in the perpendicular direction [1 0 0]. In the presence of an increasing amount of acrylic‐acid‐functionalized polystyrene particles, the crystals became shorter and wider. This decrease in the aspect ratio was correlated with the adsorption process of the polymer on the growing sites of the zinc oxide crystals, which was found to follow a Langmuir isotherm model at lower concentrations. The functionalization of the latex particles with groups showing a strong affinity towards the {0 0 1} facets of the growing crystals, such as carboxylates and phosphates, resulted in very peculiar nanostructures as a consequence, in a limiting case, of the blocking of the growth in the [1 0 0] direction. The polymer could be effectively removed by calcination. The crystallite size obtained with the Scherrer formula for the different zinc oxide morphologies ranged from 40 to 100 nm.^67^, ^69^ Interestingly, while the presence of latex particles affected significantly the spectroscopic features of the materials‐mainly by reducing the defect‐related visible photoluminescence,^70^ the long range crystalline order remained essentially undisturbed.^69^

## 5 Conclusions and Outlook

Nanodroplets in a miniemulsion system are able to confine spaces in which chemical processes can take place. This Minireview has revised the advancements on the use of both miniemulsion themselves and materials prepared in miniemulsion to confine and control crystallization processes of metal oxides.

The different approaches for preparing metal oxide nanoparticles from miniemulsions of metal precursors have been classified in three groups: I) “two‐emulsion methods”, based on the coalescence between droplets containing the metal precursor and the precipitating agents; II) external addition of the precipitating agent to an emulsion of the metal precursor; and III) emulsions in which both metal precursor and precipitating agent are simultaneously contained in the dispersed phase. The latter case also includes the so‐called single‐source precursors, which are compounds that are sources of both the metal and the oxygen. Peroxo‐complexes are typical examples of single‐source precursors.

The article has also reviewed the confinement of the formation of metal oxide nanocrystals on the surface of polymer particles, which are previously prepared by miniemulsion polymerization. Notably, the obtained materials show potentiality as heterogeneous catalysts and can also encapsulate further components in the core. Finally, the last section has demonstrated that polymer particles prepared by miniemulsion polymerization can also be used as controlling agents for the formation of nanostructured metal oxides.

Among the significant advantages of the miniemulsion technique, we find its versatility to produce a variety of systems of different‐and even hybrid‐nature, and to allow the synthesis of certain phases under milder conditions than required by other techniques (lower temperature and lower pressure). This latter point, the focus of current investigations, is mainly a result of the droplet confinement. However, as with all techniques, miniemulsion has also limitations. One point to take into account is the limited control of shape by this method. Another not negligible limitation, which can be of relevance for certain applications, especially where high purity of the inorganic phase is relevant (e.g., electronic applications), is the presence of surfactant in the final material. The removal of organic matter by calcination may let behind a carbonaceous residue, even under an oxidative atmosphere, and removal with organic solvents is not always completely efficient. Accordingly, in the context of metal oxide synthesis, miniemulsion is rather suited for applications in fields in which the presence of remaining surfactant or organic matter is not decisive. Nevertheless, it needs to be mentioned that this apparent disadvantage can turn into an advantage when dispersion in an organic medium is useful (e.g., in some cases of heterogeneous catalysis, in cosmetics, in industrial pigments, etc.), because metal oxides prepared in miniemulsion are typically “born” in an organic solvent, so that further functionalization becomes unnecessary or can by directly implemented during the miniemulsion steps.

Currently, the most promising direction for on‐going and future work appears to be the combination of miniemulsions with other techniques, such as hydrothermal synthesis. Exposing miniemulsion droplets to pressure may open new possibilities in the preparation of crystalline phases not reachable by conventional miniemulsions.

## Conflict of interest

The authors declare no conflict of interest.

## Biographical Information

*Rafael Muñoz‐Espí received his doctoral degree in Chemistry in 2006 from the Johannes Gutenberg University of Mainz*, *working at the Max Planck Institute for Polymer Research (MPIP) with Prof. G. Wegner. After two years as a postdoctoral associate at Stony Brook University, New York, he returned to Mainz, where he was a group leader at the MPIP from 2009 to 2015. Since September 2015, he has been a faculty member at the Institute of Materials Science of the University of Valencia (ICMUV). His research interests include mineralization processes, the synthesis of polymer and hybrid nanoparticles, crystallization in colloidal systems, and the study of the interaction of polymers with inorganic matter*.

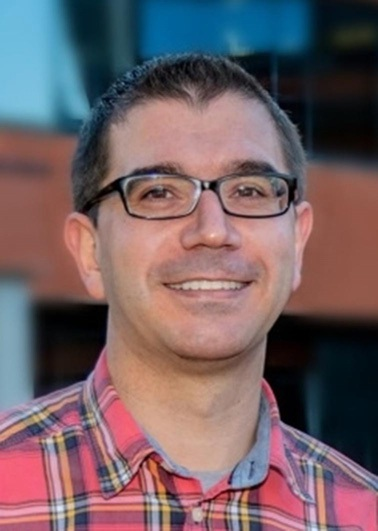

## Biographical Information

*Katharina Landfester joined the Max Planck Society in 2008 as one of the directors of the Max Planck Institute for Polymer Research. She received her doctoral degree in Physical Chemistry after working with Prof. H.W. Spiess at the Max Planck Institute for Polymer Research. In 1996, she moved for a doctoral stay at Lehigh University. She returned to Germany in 1998, working at the Max Planck Institute of Colloids and Interfaces in Golm. There, she led the miniemulsion group. In 2003, she accepted a chair (C4) of Macromolecular Chemistry at the University of Ulm. She has published more than 700 peer‐reviewed papers and holds 30 patents*.